# Cross-subject EEG driving fatigue detection: early personal spectral reference and causal long-sequence residual encoding

**DOI:** 10.3389/fnins.2026.1997832

**Published:** 2026-10-30

**Authors:** Songdi Gao, Lanlan Lv

**Affiliations:** 1 School of Computing and Information Science, Fuzhou Institute of Technology, Fuzhou, China; 2 School of Design Art, Xiamen University of Technology, Xiamen, China

**Keywords:** cross-subject, driving fatigue, early personal spectrum, EEG, residual encoding, vigilance estimation

## Abstract

**Background:**

In cross-subject electroencephalography (EEG)-based driving-fatigue detection, most existing decoders are designed around second-scale short windows, whereas fatigue is a slow drift; short-window spectral features provide little direct evidence of that slow drift, limiting fatigue decisions. More critically, each individual’s spectral baseline level and fatigue-change pattern differ, so simply lengthening the window still leaves models struggling to predict across individuals from observed spectral features. This work aims to enable cross-subject fatigue decisions under shared parameters, without per-driver specialized models.

**Methods:**

We propose early personal spectral reference with causal long-sequence residual encoding: an early personal spectral reference is estimated only from a short session-opening spectral window; subsequent spectral observations are mapped into residual space; state-conditioned temporal modeling accumulates and updates a residual state over a long causal sequence; and fatigue is read out from the terminal state.

**Results:**

On leave-one-subject-out binary classification of the Multimodal Phenotyping Dataset of Driving Fatigue (MPD-DF), five-seed (2026–2030) macro-averaged F1 is 0.655 ± 0.005 (0.660 at seed 2026), outperforming the best matched-protocol absolute-spectrum baseline (≈0.587). On continuous vigilance regression of the SEED Vigilance dataset (SEED-VIG) under a subject-LOSO protocol, root mean square error (RMSE) is 0.209 ± 0.005 under 5Bands and 0.200 ± 0.003 under 2 Hz, both below matched-protocol baselines.

**Conclusion:**

An early personal spectrum estimated from a short opening window can reduce interference from intrinsic individual spectral level in fatigue decisions and provides a practical premise for cross-subject fatigue decoding. Accumulating and updating residual state over a causal long sequence in residual space further improves cross-subject decisions. Under shared parameters without per-driver models, the framework outperforms matched absolute-spectrum baselines on both fatigue classification and vigilance regression.

## 1 Introduction

Driving fatigue is a major risk factor for road traffic safety and public health; its neural and behavioral mechanisms have been reviewed extensively (Lal and Craig, 2001). Electroencephalography (EEG) can reflect fluctuations of central alertness in near real time and has therefore been widely used in fatigue-detection experiments and algorithm research (Hussein et al., 2023; Zhang et al., 2025; Zheng and Lu, 2017). Inter-subject variability in EEG typically exceeds intra-subject fluctuation (Saha and Baumert, 2020), making cross-subject generalization a central challenge. Meanwhile, most existing models are designed around fixed temporal scales (e.g., 1 s or 3 s short windows) and struggle to accommodate cognitive-state tasks with different drift rates. Concretely, fast-drift tasks (e.g., motor-imagery BCI) evolve on a second-level timescale, for which local spatiotemporal patterns already provide discriminative information (Lawhern et al., 2018; Schirrmeister et al., 2017); fatigue is a slow drift whose signal accumulates slowly over minutes and cannot be observed directly inside a short window. Under cross-subject settings, individual differences further amplify this difficulty and hinder generalization to new drivers (Liu et al., 2020; Saha and Baumert, 2020; Zhang and Wu, 2020). Focusing on fatigue as a canonical slow-drift task, this work investigates how to introduce an explicit individual reference into the model design and accumulate slow-drift evidence over a causal long sequence, so as to reduce interference from identity features in fatigue decisions and improve cross-subject generalization.

EEG-based driving-fatigue methods have evolved from early “hand-crafted features + shallow classifiers” pipelines toward a landscape that also includes end-to-end deep architectures. Early studies typically followed a “extract spectral features, then feed a discriminative model” paradigm: compact spectral representations such as differential entropy or power spectral density characterize instantaneous alertness (Hussein et al., 2023), and shallow classifiers such as support vector machines complete the decision (Cortes and Vapnik, 1995). Convolutional, recurrent, and graph neural networks have since substantially strengthened representation capacity (Cui et al., 2023; Ding et al., 2025; Huang et al., 2025); end-to-end learning from raw multichannel EEG has likewise been widely explored to jointly optimize feature extraction and classification mapping and to avoid information loss from hand-crafted design (Cui et al., 2022a; Schirrmeister et al., 2017). For cross-subject settings, domain adaptation and attention-weighted transfer learning are also widely adopted to mitigate distribution shift from individual differences (Chen et al., 2021; Zhang et al., 2025; Zhao et al., 2021). Nevertheless, these advances preserve the same temporal design –whether hand-crafted spectral features or end-to-end encoders are used, decisions remain based on absolute spectral segments inside short windows (Perkins et al., 2023; Zhang et al., 2025). In other words, feature extraction has progressed substantially, yet decisions still rest on absolute spectra, with two limitations: (i) without an individual reference, models cannot tell whether spectral change stems from individual differences or from fatigue; (ii) without long-horizon temporal accumulation, minute-scale slowly accumulating deviation evidence cannot be aggregated into a reliable decision. This limitation is especially acute under the fast-/slow-drift mismatch –when only second-scale absolute spectra are available, slow-drift fatigue cues are easily masked by identity differences.

Recently, hybrid convolution–Transformer–recurrent architectures have strengthened modeling of fatigue dynamics via local filtering, long-range dependency, and temporal state evolution (Wang et al., 2025); for cross-subject settings, domain alignment and attention-weighted transfer expand applicability to new drivers by suppressing negative transfer and aggregating multi-source knowledge (Jayaram et al., 2016). These advances significantly improve the expressivity and cross-subject usability of EEG fatigue decoders (Wang et al., 2025). Structural limitations remain. On one hand, applying stronger temporal interaction (e.g., self-attention) directly to physiological time series may face a structural mismatch between “decentralized interaction” and the “centralized coordination” that signals often require –medical time series are frequently driven by a central mode, whereas standard attention can dilute that central pattern (Yu et al., 2026). On the other hand, although such methods can mitigate cross-subject distribution shift, they need not structurally separate “who the individual is” from “whether the individual is fatigued.”

Based on these limitations, this paper focuses on the following research questions:

RQ1: Under cross-subject settings, can a personal spectral reference estimated solely from a very short session-opening window effectively reduce interference from individual differences in fatigue decisions?

RQ2: Does causal sequence accumulation on the residual trajectory capture slow-drift fatigue evidence better than using only the instantaneous residual? What causal-window length is required?

RQ3: Does the framework behave consistently across discrete classification and continuous regression?

Correspondingly, we hypothesize:

H1: After subtracting the opening personal spectral reference, cross-subject fatigue decision performance is substantially better than absolute-spectrum baselines.

H2: Causal sequence accumulation on the residual trajectory improves task performance relative to using only the instantaneous residual; gains plateau after a moderate window length rather than growing indefinitely with window length.

H3: Classification and regression depend on sequence accumulation to a similar degree.

Note that long sequence/long window” here is relative to the second-scale short windows (about 1–3 s) commonly used by existing EEG decoders:long’ ’ denotes a relative temporal span, not an absolute hour-scale window.

To address these research questions, we propose a two-component framework for slow-drift fatigue: early personal spectral reference and causal long-sequence residual encoding. The former estimates the early personal spectral reference from a short frozen opening history only, and maps subsequent observations into the residual space relative to that reference –structurally attenuating the temporally stable individual spectral level and emphasizing fatigue-related deviation as the premise of cross-subject decisions. The latter performs within-frame feature reorganization, conditioned temporal modeling, and persistent state updates in sequence on causal long windows of the residual trajectory, accumulating slow-drift evidence into a compact representation for classification or continuous vigilance estimation. Parameters are shared across subjects; neither per-driver model copies nor additional domain alignment are required.

Our main contributions are as follows:

1. We demonstrate the necessity of early personal spectral reference for cross-subject fatigue decisions: an early personal spectral reference estimated solely from the session-opening frozen window can effectively suppress identity interference in absolute spectra and reduce mixing of individual level with fatigue signals under shared parameters without domain alignment.
2. We propose and validate causal sequence residual encoding: slowly changing deviation evidence is accumulated within a causal window of the residual trajectory into a compact state for decision making. Relative to using only the instantaneous residual without sequence accumulation, sequence encoding yields gains on both classification and regression; sensitivity analysis further shows that gains plateau after a moderate window length rather than increasing indefinitely with length.
3. We verify generality across tasks and datasets: on MPD-DF cross-subject binary classification and SEED-VIG continuous vigilance regression, the framework substantially outperforms matched absolute-spectrum baselines and representative domain-alignment/transfer methods. Classification and regression share the same backbone and differ only in the readout head, indicating that the framework does not rely on task-specific feature design. Ablations further reveal differing dependence on accumulated representations across tasks, informing subsequent task adaptation.

## 2 Related work

### 2.1 CNN-based EEG decoding and fatigue recognition

CNN-based EEG decoding established an important foundation for end-to-end learning from raw or near-raw EEG. Early work such as DeepConvNet and ShallowConvNet showed that discriminative spatiotemporal patterns can be learned directly from raw inputs, reducing dependence on hand-crafted feature engineering (Schirrmeister et al., 2017). EEGNet further improved compactness via depthwise and separable convolutions, enabling efficient spatiotemporal representation learning and becoming a common baseline across paradigms (Lawhern et al., 2018). Spatiotemporal CNNs for driving fatigue (e.g., ESTCNN) explicitly model drowsiness-related dynamics along channel–time structure (Gao et al., 2019); compact/interpretable CNNs demonstrated learnable shared drowsiness patterns in cross-subject driver drowsiness recognition, with emphasis on visualization and parameter efficiency (Cui et al., 2022a). Signal-decomposition ensemble CNNs, dense convolutional Transformers, and edge-oriented lightweight graph convolutions further expand capacity along multi-scale decomposition, coarse-to-fine temporal dynamics, and deployment constraints (Ding et al., 2025; Huang et al., 2025; Li et al., 2023). Under a spectral-feature regime, VIGNet takes differential entropy as input and uses deep convolutions for vigilance/drowsiness estimation, exemplifying the mainstream “spectral representation + CNN” recipe for fatigue (Ko et al., 2020). Graph-convolution models such as DGCNN, though often originating from emotion EEG, are frequently included in fatigue comparison tables to capture channel topology (Song et al., 2020). Recent end-to-end spatiotemporal EEG work further advances this line: EmoDLNet learns spatiotemporal representations directly from raw EEG with multi-scale 2D/1D convolutions and hierarchical recurrent units, validating real-time inference in cross-subject emotion recognition and explicitly discussing deployment metrics such as inference latency (Geda et al., 2026a). Overall, CNN routes substantially strengthen local spatiotemporal and channel–band expressivity; such decoders typically decide on absolute observations (raw segments or absolute spectra), so fatigue-related changes can be masked by intrinsic individual spectral level in cross-subject fatigue tasks.

### 2.2 Attention and hybrid architectures for EEG decoding

Early attention served mainly as an auxiliary module for selective feature enhancement; it has more recently entered the backbone, especially in CNN–Transformer/CNN–LSTM hybrids. For driving fatigue and vigilance estimation, convolution–Transformer–recurrent hybrids jointly perform local filtering, long-range dependency modeling, and temporal state evolution to predict continuous fatigue indices such as PERCLOS (Dinges and Grace, 1998; Wang et al., 2025); dynamical encoder–decoder and filtering frameworks likewise estimate instantaneous drowsiness from EEG (Arefnezhad et al., 2022); dense convolutional Transformers further compress long-range dependence into a unified backbone (Ding et al., 2025). In cross-subject settings, attention is also used to weight-aggregate multi-source models (e.g., ATR-EEG (Zhang et al., 2025)) to mitigate individual differences and suppress negative transfer (Zhang et al., 2025). Concurrently, medical time-series studies note that physiological signals often exhibit shared global/centralized structure, whereas decentralized self-attention does not inherently provide such a core reference, yielding a potential inductive-bias mismatch (Yu et al., 2026). Thus, although hybrid architectures enlarge modeling capacity and cross-subject adaptation, their decisions still largely rest on absolute observations, and temporal interaction mechanisms remain in potential tension with the centralized organization of physiological signals. More importantly, such architectures still rarely address two structural requirements of slow-drift fatigue: (i) fatigue evidence accumulates slowly over time, yet existing decoders still decide from second-scale short windows (Zhang et al., 2025; Perkins et al., 2023); recent long-sequence vigilance-estimation work has noted that slow accumulation of fatigue and short-term vigilance fluctuations call for different temporal modeling strategies, which existing methods rarely address effectively (Song, 2026; Huangfu et al., 2026); (ii) online deployment requires causality, whereas bidirectional models can access the full session during training and conflict with streaming inference (Niu et al., 2026). Slow-drift fatigue therefore needs cumulative encoding of sequences under causal constraints, rather than applying bidirectional temporal interaction directly to short-window absolute spectra. The causal sequence residual encoding in this paper follows this rationale, accumulating fatigue-related deviations on residual trajectories via causal convolution and causal weighted pooling.

### 2.3 Driving-fatigue EEG datasets and task spectrum

EEG studies of driving fatigue/drowsiness form a relatively clear task spectrum. Early work combined band-power spectral features with shallow classifiers (Jap et al., 2009; Lal and Craig, 2001); psychophysiological and neurophysiological reviews organize measurement frameworks for driving fatigue, mental workload, and drowsiness (Borghini et al., 2014; Lal and Craig, 2001); systematic reviews cover methods, modalities, and deployment challenges (Ajayi et al., 2025; Hussein et al., 2023; Perkins et al., 2023). Among public benchmarks, SEED-VIG advanced continuous vigilance estimation from EEG (and forehead EOG) and subsequent deep methods (Zheng and Lu, 2017); multi-channel sustained-attention driving EEG (Cao et al., 2019) and multimodal driving-fatigue phenotyping data (MPD-DF) (Li et al., 2026) support cross-center validation. On the hardware side, low-cost headsets and behind-the-ear wearable EEG discuss deployment feasibility (LaRocco et al., 2020; Nguyen et al., 2023), further underscoring the need for algorithms that remain usable under cross-subject settings. These datasets and deployment discussions cover supervision forms from discrete fatigue grading to continuous vigilance estimation and provide a common basis for cross-subject algorithm evaluation.

### 2.4 Cross-subject evaluation and personalization

Cross-subject work mostly adopts *post-hoc* correction via domain alignment/transfer learning and seldom establishes a clean individual reference at the protocol level; meanwhile, minute-scale cues of slow-drift tasks are often overshadowed in model inductive bias by fast-drift or short-window designs. Addressing cross-subject failure, BCI transfer-learning surveys and inter-/intra-subject variability analyses argue that cross-person generalization must be posed explicitly rather than assuming i.i.d. subjects (Jayaram et al., 2016). Domain adaptation, manifold-embedded transfer, and inter-subject transfer have been widely explored for mental fatigue/driver mental-state tasks (Cui et al., 2022b; Liu et al., 2020; Zhang and Wu, 2020); plug-and-play domain adaptation, multi-source marginal distribution alignment, and cross-subject domain generalization on emotion EEG likewise treat “cross-person” as a distribution-shift problem (Chen et al., 2021; Wang et al., 2024; Zhao et al., 2021). Recent multimodal work similarly applies adversarial domain adaptation to learn subject-invariant representations –for example, combining CNN–BGRU with adversarial domain adaptation for VR shopping-preference prediction and outperforming unimodal baselines under LOSO evaluation (Geda et al., 2026b). As preprocessing, Euclidean alignment (EA) whitening of covariances shrinks second-order inter-subject differences and is often a strong default on covariance-rich motor-imagery features (He and Wu, 2020; Wu, 2025); CORAL and DANN exemplify second-order statistical alignment and adversarial alignment, respectively (Ganin et al., 2016; Sun and Saenko, 2016). Zero-calibration cross-subject drowsiness detection, manifold-level augmentation, and test-time adaptation further indicate that calibration-free settings are a central deployment concern (Jang et al., 2025; Kim et al., 2023; Paulo et al., 2021). The above lines acknowledge individual differences as a primary obstacle to cross-subject fatigue decoding and commonly respond with normalization or calibration. Two gaps remain: (i) calibration itself incurs cost in time and data and is inconvenient for deployment, motivating recent calibration-free or domain-specific normalization studies (Jang et al., 2025; Kim et al., 2024; Kim et al., 2025); (ii) the reference is often not obtained cleanly –most methods estimate mean/variance from the full session so that late fatigue data enter the statistics, and the few studies that use an opening baseline typically use it only for model selection rather than as a subtractive reference (Wei et al., 2018). Inspired by these attempts, we move the reference further forward to the session opening: estimate a personal spectral reference once at the opening and then freeze it, so that subsequent signals enter the decoder as deviations relative to that reference. This paper addresses that gap by combining early personal spectral reference with causal long-sequence residual encoding to improve generalization of cross-subject slow-drift fatigue decisions.

## 3 Materials and methods

This section details the proposed cross-subject driving-fatigue detection framework (illustrated in Figure 1), whose core comprises early personal spectral reference and causal long-sequence residual encoding. We first formalize the differential-entropy problem and the early personal spectral reference protocol, then describe residual-trajectory construction, modular single-pass long-sequence encoding, and the classification/regression readout heads.

**FIGURE 1:**
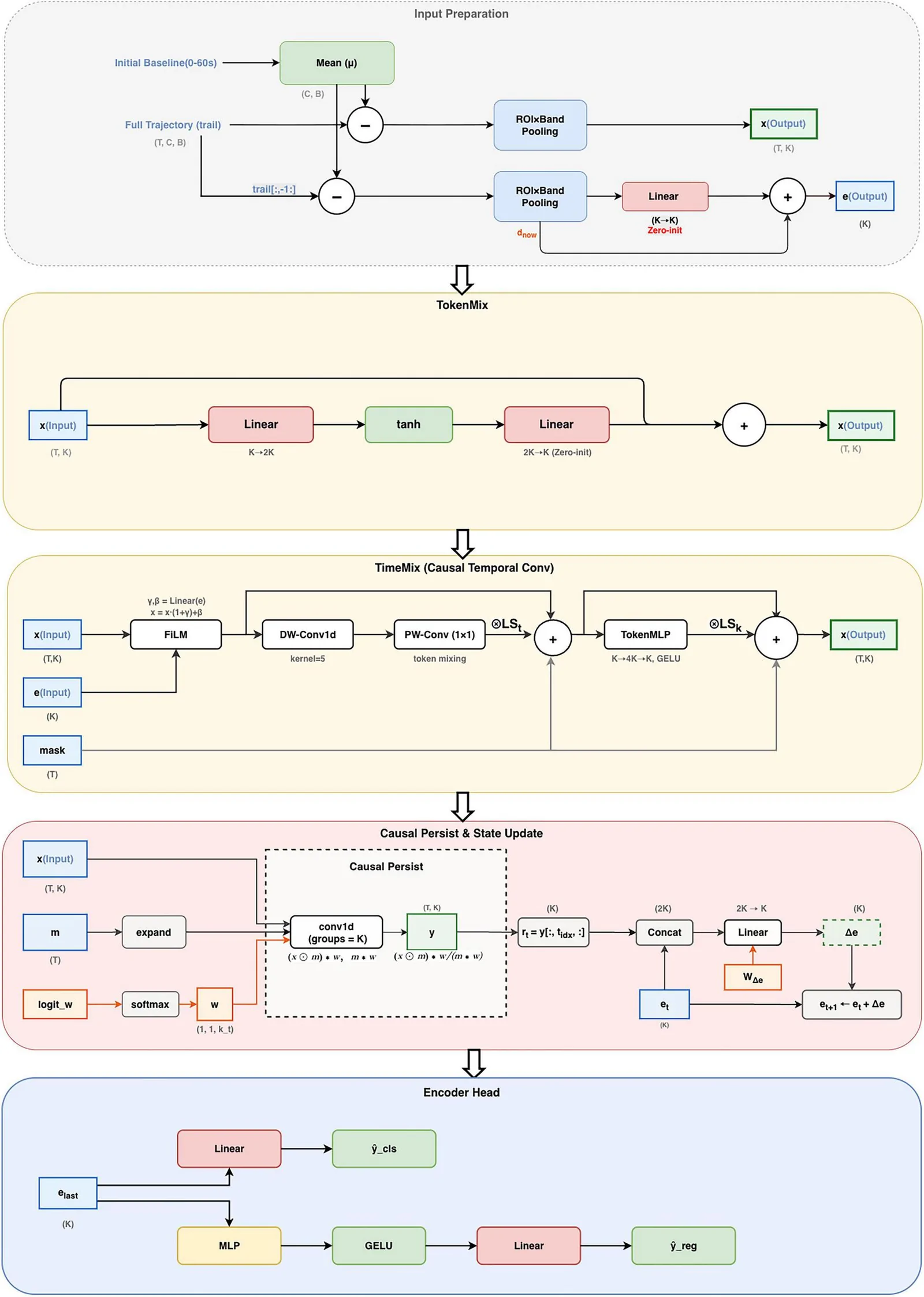
Schematic of early personal spectral reference and causal long-sequence residual encoding. An opening freeze window estimates the early personal spectral reference μ; subsequent differential-entropy (DE) frames form a residual trail relative to μ, encoded by within-frame token reorganization, causal temporal mixing (modulated by residual state *e*), causal persistent readout, and a state update. The proposed method reads out from the updated residual state *e*; *d*_*now*_ is the pre-encoding instantaneous residual used in section “4.3 Ablation studies” ablations.

### 3.1 Problem formulation and early personal spectral freeze protocol

Consider a differential-entropy (DE) sequence from one driving session. Within each frequency band, EEG is commonly modeled as Gaussian *N*(*m*,σ^2^), and differential entropy is given by Equation 1:


$$\mathrm{DE}=\frac{1}{2}\,\log\,\left(2\,\pi \,e\,\sigma^{2}\right)$$ (1)

which follows from the closed-form differential entropy of a normal distribution (Duan et al., 2013). The main configuration uses DE-LDS smoothed spectral features consistent with SEED-VIG (Shi and Lu, 2010; Zheng and Lu, 2017). Let the frame at second *t* be *F*_*t*_∈ℝ^*C*×*B*^, where *C* is the number of electrodes (*C* = 32 on MPD-DF; *C* = 17 on SEED-VIG) and *B* is the number of bands. Channels are aggregated by anatomical regions of interest (ROI) and restricted to α/β, yielding token dimension *K* (main setting *K* = 8). This choice is a compact anatomical prior and dimensionality reduction shared across subjects; empirical motivation appears in section “4.1 Preliminary analysis of the main feature setting.” Under a subject-independent/LOSO protocol, the test subject and all of their samples are excluded from training and validation.

For slow-drift fatigue we adopt a hard early-freeze protocol. Given freeze length *L* (main setting *L* = 60*s*), the opening freeze history used solely to estimate the early personal spectral reference is given by Equation 2:


$$H=\left(F_{0},F_{1},\ldots ,F_{L-1}\right)\in {\mathbb{R}}^{L\times C\times B}$$ (2)

This choice is motivated by topography of spectral residuals in the opening window (section “4.1 Preliminary analysis of the main feature setting”) and freeze-length sensitivity [section “4.3.5 Freeze-length *L* sensitivity (MPD-DF)”]. We define μ as the early-session slow spectral intercept (early personal spectral reference), not as a “pure alertness anchor” –even if the opening window already contains partial slow drift, subtracting μ removes a relatively stable individual offset rather than guaranteeing removal of an alert physiological state. The opening window *H* is used only to estimate μ: it does not enter the causal trail, carries no supervised labels, and therefore contributes no label-driven gradients through the encoder. From second *L* onward (*t*≥*L*), each eligible subsequent time is an independent prediction target–sampled every 10 *s* on MPD-DF (fatigue changes slowly; adjacent seconds are highly redundant) and every second on SEED-VIG. Later frames must not write back into the early personal spectral reference, preventing late slow-drift contamination of the reference.

The goal is to learn a shared-parameter map *f*_θ_ that predicts fatigue class or continuous vigilance for *t*≥*L*. Unlike scoring absolute frames *F*_*t*_ directly, the model makes decisions in the residual space relative to the early intercept. Slow-drift evidence is accumulated inside each causal trail window of length *T* (section “3.3 Causal long-sequence residual encoding;” main settings *T* = 600*s* on SEED-VIG and *T* = 3600*s* on MPD-DF); the compact state *e* is not carried across supervised times as a session-long recurrent memory. Instead, each prediction uses a right-aligned causal trail of length *T* (sliding with *t*) for within-window aggregation; this is within-window persistence, not session-level online recurrence across supervised times.

### 3.2 Early personal spectral reference and residual trajectory

Figure 2 illustrates the temporal relation between the opening freeze window *H* and subsequent sliding causal trails: supervised times fall at causal-window ends; predictions at different times are independent and later frames are never written back into μ.

**FIGURE 2:**
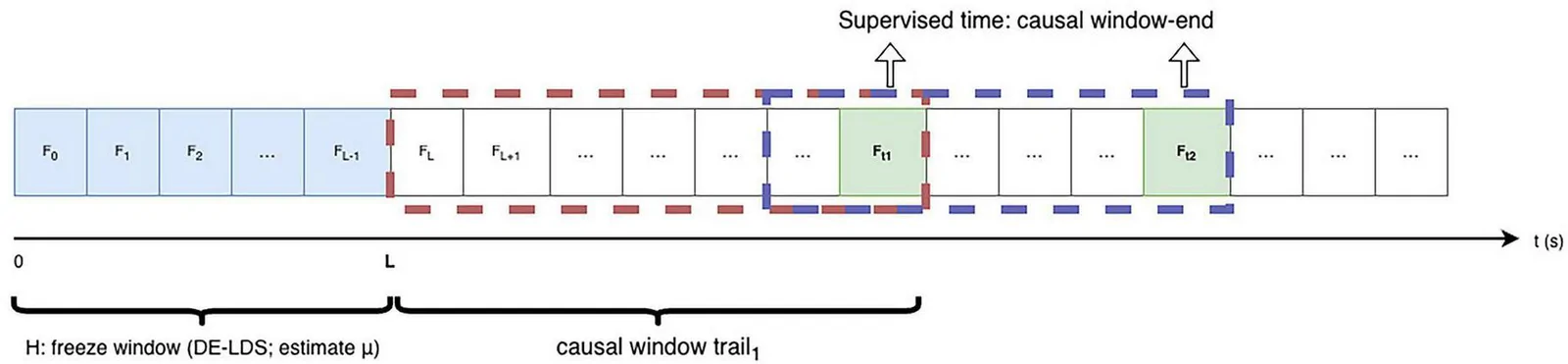
Opening freeze window and sliding causal trails. The first *L* seconds of DE-LDS frames form freeze window *H*, used only to estimate the early personal spectral reference μ and then locked. From second *L* onward, each supervised time *t* (e.g., *t*_1_, *t*_2_ in the figure) corresponds to a causal *trail* right-aligned at that time, which is encoded and read out independently in residual space relative to μ.

Our μ follows baseline-calibration practice: an early reference is used to center subsequent observations (Yu et al., 2026). Conceptually, for *t*≥*L* each frame may be written $F_{t}=\mu_{i\,d}+\Delta \,F_{t}^{f\,a\,t\,i\,g\,u\,e}+\varepsilon_{t}$, where μ_*id*_ is a slowly varying individual mean of DE frames, $\Delta \,F_{t}^{f\,a\,t\,i\,g\,u\,e}$ is fatigue-related change, and ε_*t*_ is noise. We set μ as shown in Equation 3:


$$\mu =\frac{1}{L}\,\sum_{\tau =0}^{L-1}F_{\tau }\in {\mathbb{R}}^{C\times B}$$ (3)

Given a causal trail window *trail*∈ℝ^*T*×*C*×*B*^ of length *T* with mask *m*∈{0,1}^*T*^ at a supervised time, form the residual trail and apply ROI×band pooling (elementwise mean over electrodes within each ROI and over the retained α/β bands) as defined in Equation 4:


$$X=t\,r\,a\,i\,l-\mu ,X={\mathrm{Pool}}_{\mathrm{ROI}\times \mathrm{band}}\,\left(X\right)\in {\mathbb{R}}^{T\times K}$$ (4)

Let *F*_*t*_ be the frame at the current supervised time, identified with the last valid frame of *trail* under the mask (right-aligned full windows: the last frame of *trail*). The instantaneous residual –denoted *d* in Equation 5 and *d*_*now*_ in ablations –is the pooled residual of this current frame relative to μ, computed before any TokenMix/TimeMix rewrite of *X*:


$$d={\mathrm{Pool}}_{\mathrm{ROI}\times \mathrm{band}}\,\left(F_{t}-\mu \right)\in {\mathbb{R}}^{K}$$ (5)

Under the above convention, *d* also equals the last valid frame of the residual trail *X* prior to encoding. For every supervised time *t*, the residual state is re-initialized from *d* by an identity-started linear refinement (no carry-over of *e* from a previous supervised time) as defined in Equation 6:


e=d+ψ⁢(d) (6)

Here *ψ*:ℝ^*K*^→ℝ^*K*^ is a Linear map with weights and bias zero-initialized. At the start of training ψ ≡ 0, hence *e* ≡ *d*; training allows a mild affine correction of the initial state rather than a separate nonlinear encoder. Within the current trail only, *e* conditions TimeMix via Feature-wise Linear Modulation (FiLM) (Perez et al., 2018) and receives one update from the Persist readout (section “3.3 Causal long-sequence residual encoding);” the main method then reads the updated *e* (section “3.4 Readout head).” The key constraint is that μ may be estimated only from *H*; thereafter all modules operate on the residual sequence *X* and state *e*, realizing an absolute-to-relative transform via “early aggregation → subtractive redistribution.” If the opening window already contains partial slow drift, μ remains a valid early-session intercept; subtracting μ removes a relatively stable individual offset rather than promising to subtract a purely alert state. At deployment, each supervised time is executed independently: take the causal window ending at that time, initialize *e* from the instantaneous residual at that time, encode within the window, and read out; the next supervised time re-initializes *e* without inheriting the previous state. Thus accumulation of *e* is confined to a single prediction window and does not constitute session-level recurrence across supervised times.

### 3.3 Causal long-sequence residual encoding

Given residual trail *X*∈ℝ^*T*×*K*^ (mask *m*∈{0, 1}^*T*^) and state *e*∈ℝ^*K*^ re-initialized as in Equation 6, a single encoding pass proceeds, consistent with the architecture Figure 1, as TokenMix → TimeMix → Causal Persist → state update. Informally: TokenMix reorganizes ROI×band residual coordinates within each time step; TimeMix uses short-kernel causal convolution to capture near-second residual slopes and conditions the entire trail on *e* via FiLM; Causal Persist performs causal weighted moving averaging so that the end-time readout *r* already aggregates history inside the current window; finally *r* updates *e* once for the readout head. This is within-trail accumulation of length up to *T*, not a hidden state threaded across successive supervised times. Conditioning of the trail by *e* occurs only through FiLM, so that *X* primarily reflects the residual sequence relative to μ and its local smoothing. Terminal layers of residual branches (or increment maps) are zero-initialized so that training begins near identity and learns corrective deviations from data. Encoding rewrites *X*, so the Persist end-frame generally differs from the initializing *d*; by contrast, ablation *d*_*now*_ remains the pre-encoding residual in Equation 5.

TokenMix (within-frame token reorganization). Mix the *K*-dimensional tokens within each time step only (no cross-time mixing) to locally reorganize ROI×band residual coordinates. An MLP (*K*→2*K*, tanh, 2*K*→*K*; last layer zero-initialized) is applied per time, written back residually, and padded positions are cleared by the mask as defined in Equation 7:


X←(X+TokenMix⁢(X))⊙m (7)

TimeMix (causal cross-time mixing + state conditioning). Perform causal cross-time mixing on the residual trail, conditioned on *e*. First form FiLM parameters from *e* (γ,β∈ℝ^*K*^, broadcast over time; linear layer zero-initialized) as defined in Equation 8:


(γ,β)=L⁢i⁢n⁢e⁢a⁢r⁢(e),X←X⊙(1+γ)+β (8)

Then apply causal depthwise separable convolution: left-pad *k*_*t*_−1 frames along time (main setting kernel length *k*_*t*_ = 5, dilation 1), depthwise grouped convolution followed by 1×1 pointwise convolution yields a temporal increment; write back residually with learnable LayerScale (Touvron et al., 2021) *LS*_*t*_∈ℝ^*K*^ (per-channel multiplicative gains on residual branches; input-independent, initialized to 1) and remask with *m*. A subsequent Token-MLP (*K*→4*K*→*K*, GELU; last layer zero-initialized) is written back with *LS*_*k*_∈ℝ^*K*^ as defined in Equations 9, 10:


$$X\leftarrow X+{\mathrm{LS}}_{t}⊙\mathrm{PW}\,\left(\mathrm{DW}-{\mathrm{Conv}}_{\mathrm{causal}}\,\left(X\right)\right),X\leftarrow X⊙m$$ (9)

$$X\leftarrow X+{\mathrm{LS}}_{k}⊙\mathrm{TokenMLP}\,\left(X\right),X\leftarrow X⊙m$$ (10)

*LS*_*t*/*k*_ are residual-branch gains, distinct from FiLM (γ,β) produced by *e*. This branch is not self-attention: temporal inductive bias is supplied by causal convolution, while *e* conditions only via FiLM –a clear division of labor.

Causal Persist (causal persistent readout). Compress the mixed trail into a history-aware valid end-time readout *r*. Apply grouped causal weighted pooling to masked *X*: learnable kernel logits (trained end-to-end) are softmax-normalized to a probability kernel *w*∈Δ^*k*_*t*_−1^ (shared across *K* tokens; main setting *k*_*t*_ = 5, matching TimeMix). The logits are initialized to zero, so at the start of training *w* is uniform (a moving average), providing a stable smoothing prior before task-specific weighting is learned; after left padding, grouped convolution with mask normalization yields a smoothed trail *Y*∈ℝ^*T*×*K*^ as defined in Equation 11:


$$Y=\frac{{\mathrm{Conv1d}}_{\mathrm{causal}}\,\left(X⊙m;w\right)}{{\mathrm{Conv1d}}_{\mathrm{causal}}\,\left(m;w\right)+\varepsilon },r=Y_{t_{i\,d\,x}}\in {\mathbb{R}}^{K}$$ (11)

where *t*_*idx*_ is the last valid time under the mask and ε is a numerical stabilizer. Because the convolution is causal, *Y*_*t*_*idx*__ already mixes preceding frames inside the trail; taking the end index is the online readout of that smoothed history, not a claim that only the raw last frame matters. From the start of training this operator is a smoother (a uniform kernel is a moving mean), not an identity residual around the current frame.

State update. Concatenate readout *r* with the current state and map to an increment (linear layer zero-initialized) using Equation 12:


$$\Delta e=W_{\Delta \,e}\text{[}r;e],e\leftarrow e+\Delta e$$ (12)

where [*r*;*e*]∈ℝ^2*K*^ and *W*_Δ*e*_:*R*^2*K*^→*R*^*K*^. Thus, for the present supervised time only, *e* receives one Δ*e* update from the initialization in Equation 6 and then enters the readout head (section “3.4 Readout head).” At the next supervised time, *e* is again re-initialized from that time’s *d* via Equation 6.

### 3.4 Readout head

After encoding, the main method uses only the final residual state *e* for readout as shown in Equation 13:


$$\hat{y}=H\,e\,a\,d\,\left(e\right)$$ (13)

Both tasks share the same backbone and differ only in the head: (i) MPD-DF binary classification uses a linear head *Linear*(*K*→2); (ii) SEED-VIG continuous regression uses a two-hidden-layer MLP (hidden width 128, ReLU, Dropout) producing a scalar vigilance value. This asymmetry follows from the tasks: binary classification is already well served by a linear decision surface, whereas continuous regression needs additional nonlinear capacity to capture PERCLOS dynamics [see section “4.3.4 Regression-Head Capacity (Linear vs MLP)].”

### 3.5 Training objective

Classification uses cross-entropy (uniform class weights in the main setting) as defined in Equation 14:


$$L=-\sum_{t\in T}l\,o\,g\,p_{\theta }\,\left(y_{t}|H,{\mathrm{trail}}_{t}\right)$$ (14)

where *T* = {*t*:*t*≥*L*} (further subsampled every 10 *s* on MPD-DF; section “3.6 Experimental setup).” Regression replaces the loss with mean squared error (MSE) matched to vigilance labels. Both ends use AdamW (Loshchilov and Hutter, 2019); hyperparameters and model selection are given in section “3.6 Experimental setup.”

### 3.6 Experimental setup

We evaluate on two public driving-fatigue EEG benchmarks covering the two canonical task settings: MPD-DF is binary alert/fatigue classification; SEED-VIG is continuous PERCLOS (Dinges and Grace, 1998) regression under both feature settings below (summarized in Table 1). All experiments are subject-independent: train/val/test splits follow subjects (or sessions); test subjects and all of their samples are excluded from training and model selection. Controls and the proposed method share the same features, splits, and supervised time set; they differ in model structure and input protocol –controls decide on absolute frames *F*_*t*_, whereas we estimate μ from the early frozen window and encode residual trails causally over long sequences.

**TABLE 1:** Dataset overview (sample counts are supervised items under the protocol).

Dataset	Subject	Sample	Channel	Feature setting
MPD-DF	50	36,195	32	DE-LDS, 5 bands
SEED-VIG-5Bands	23	20,355	17	DE-LDS, 5 bands
SEED-VIG-2 Hz	23	20,355	17	Official 2 Hz spectra (not DE-LDS)

PERCLOS (percentage of eyelid closure over time) is a standard vigilance metric (Dinges and Grace, 1998); DE-LDS denotes differential entropy features smoothed by a linear dynamical system (Shi and Lu, 2010).

#### 3.6.1 Datasets and preprocessing

(1) MPD-DF (Li et al., 2026) is a multimodal driving-fatigue phenotyping dataset. Raw labels include alert, fatigue grades 1–4, and QC artifact codes 8/9. We map to binary labels: alert → 0; grades 1–4 merged → 1; codes 8/9 discarded. Features are channel-wise DE smoothed by LDS (Shi and Lu, 2010) (DE-LDS), shape 32×5 per second. Evaluation uses all-50 LOSO (50 folds; each subject is held out once; within-fold validation is split from training subjects; the test subject never enters training or selection). With *L* = 60, times *t*≥*L* are used for train/val/test and sampled every 10 *s*. Splits and sampling are identical for controls and the proposed method.

(2) SEED-VIG (Zheng and Lu, 2017) is a public vigilance-estimation benchmark with continuous PERCLOS (Dinges and Grace, 1998) labels. We evaluate on two feature subsets: 5Bands uses DE-LDS smoothed five-band features (17×5), consistent with MPD-DF’s DE-LDS pipeline; 2 Hz uses the official SEED-VIG 2 Hz spectral tensors (not DE-LDS). Evaluation follows leave-one-subject-out (LOSO): 23 sessions from 23 subjects; each fold holds out all sessions of one subject for testing, and the remaining subjects’ sessions are used for training and validation; no data from the test subject appear in training or model selection. Within training segments we still apply early freeze: the opening *L* = 60*s* estimates μ only, so labeled train/val times start at *t*≥*L*. Controls and our variants share one parameter set under this split, predict independently for *t*≥*L*, and use early *H* only to estimate the early personal spectral reference μ.

#### 3.6.2 Baselines

We compare against the following external decoders under the same protocol. Some baselines are implemented via LibEER (Liu et al., 2025); only input/output dimensions are adapted. MPD-DF and SEED-VIG share the same backbone list with task-specific heads.

- SVM-RBF/SVR (Cortes and Vapnik, 1995; Drucker et al., 1997): RBF-kernel SVM as a shallow control; binary SVM on MPD-DF and SVR on SEED-VIG; inputs are flattened absolute DE frames.
- MLP: two-hidden-layer fully connected network on flattened DE frames; a simple deep control without structured inductive bias.
- EEGNet (Lawhern et al., 2018): compact CNN with depthwise separable convolutions; reproduced on absolute DE frames.
- ESTCNN (Gao et al., 2019): spatiotemporal CNN for driving fatigue along channel–time structure.
- VIGNet (Ko et al., 2020): deep CNN on DE for driver vigilance/drowsiness; “spectral features + CNN.”
- DGCNN (Song et al., 2020): dynamical graph CNN treating electrodes as nodes; proposed on emotion EEG, often used as a fatigue/vigilance control backbone.
- DBN (Zheng and Lu, 2015): deep belief network of stacked RBMs; a classic spectral-feature deep baseline.
- GCBNet (Zhang et al., 2022): graph convolutional broad network combining graph convolution with broad learning.
- CDCN (Gao et al., 2021): channel-fused dense convolutional network aggregating multi-layer local features along channels.
- EEG-Deformer (Ding et al., 2025): dense convolutional Transformer with a shallow CNN encoder and densely connected HCT blocks to capture coarse- and fine-grained temporal dynamics of EEG for cross-subject classification.
- TSception (Ding et al., 2023): multi-scale spatiotemporal CNN using multi-scale temporal kernels and hemisphere-asymmetric spatial kernels to extract temporal dynamics and spatial asymmetry of EEG.
- LDGCN (Huang et al., 2025): edge-oriented lightweight dual-graph convolutional network on single-channel EEG, with a baseline-drowsiness adjacency graph and adaptive pruning for driver-drowsiness monitoring on the edge.
- CORAL (Sun and Saenko, 2016): second-order statistical alignment (CORrelation ALignment) by minimizing source–target covariance discrepancy; evaluated here under a domain-generalization setting (no target-domain data in training).
- DANN (Ganin et al., 2016): adversarial domain adaptation that learns domain-invariant features via a domain discriminator; evaluated here under a domain-generalization setting (no target-domain data in training).
- MSMDA (Chen et al., 2021): multi-source marginal distribution alignment for learning cross-subject shared representations; evaluated here under unsupervised domain adaptation (unlabeled target-domain data available).

All baselines read only the current absolute frame *F*_*t*_ (no *F*_*t*_−μ) and share the same supervised time set. They are therefore matched-protocol absolute-spectrum frame decoders (not matched input capacity), not long-sequence models. Isolating the contribution of early −μ under matched long-sequence encoding is reserved for the w/o Early Baseline ablation in section “4.3 Ablation studies,” rather than mixing that control into the main comparison tables.

#### 3.6.3 Implementation details

All experiments ran on a server with an NVIDIA GeForce RTX 4090. Across datasets, structural hyperparameters of the long-sequence residual encoder are fixed; only the readout head and training schedule change by task (Table 2).

**TABLE 2:** Per-dataset training hyperparameters.

Hyperparameter	MPD-DF (classification)	SEED-VIG (regression)
Max epochs	20	30
Early stopping	–	Patience = 15 (monitor val MSE)
Selection criterion	Best val Macro-F1	Best val error
Batch size	64	64

#### 3.6.4 Early-freeze protocol (structure-agnostic; shared by both ends)

For each session, the first consecutive *L* seconds of DE frames form *H* = (*F*_0_,…,*F*_*L*−1_) with main *L* = 60. The early personal spectral reference is μ = *mean*(*H*) from *H* only. Train/val/test use times from second *L* onward (*t*≥*L*), not a single end-frame of the whole session: every 10 *s* on MPD-DF; every second within the split for SEED-VIG. Absolute-spectrum baselines share the same time set but read only *F*_*t*_, not *H*. Main comparisons are therefore matched-protocol (same splits and supervised times), not matched input capacity: baselines are single-frame absolute-spectrum decoders, whereas the proposed method uses reference μ and a residual trail of length *T*. Feature standardization uses training-side statistics only.

#### 3.6.5 Fixed structural hyperparameters

Number of encoding blocks *n*_*b*_ = 1; ROI×α/β yields *K* = 8; TimeMix kernel length 5; Persist is grouped causal convolution; state update Δ*e*([*r*;*e*]) (2*K*→*K*); readout uses final *e* only. Classification head: Linear; regression head: MLP128 (ReLU, Dropout 0.3). Main reported seeds: 2026–2030.

#### 3.6.6 Optimization

Both tasks use AdamW (Loshchilov and Hutter, 2019) with weight decay 1×10^−4^ and initial learning rate 1×10^−3^. Batch size, max epochs, and selection rules follow Table 2. SVM/SVR (Cortes and Vapnik, 1995; Drucker et al., 1997) are fit once. Metrics are in section “3.7 Evaluation metrics.”

### 3.7 Evaluation metrics

Because MPD-DF and SEED-VIG are classification and regression tasks, respectively, we report task-matched metrics as mean±std over seeds/folds.

#### 3.7.1 MPD-DF classification

Decision threshold 0.5. For *N* test samples with labels *y*_*i*_ and predictions ${\hat{y}}_{i}$ as defined in Equation 15:


$$ACC=\frac{1}{N}\sum_{i=1}^{N}1\left({\hat{y}}_{i}=y_{i}\right).$$ (15)

Given class imbalance, the primary metric is macro-averaged F1 (Macro-F1). Main tables also report Accuracy and macro Precision/Recall; selection and conclusions use validation/test Macro-F1.

#### 3.7.2 SEED-VIG regression

For continuous PERCLOS (Dinges and Grace, 1998) targets *y*_*i*_ and predictions ${\hat{y}}_{i}$, we follow ATR-EEG (Zhang et al., 2025) with RMSE and MAPE(%) as defined in Equations 16, 17:


$$\mathrm{RMSE}=\sqrt{\frac{1}{N}\,\sum_{i=1}^{N}{\left(y_{i}-{\hat{y}}_{i}\right)}^{2}},$$ (16)

$$\mathrm{MAPE}\left(\%\right)=100\cdot \frac{1}{N}\sum_{i=1}^{N}\frac{\left|y_{i}-{\hat{y}}_{i}\right|}{\max\,\left(\left|y_{i}\right|,\varepsilon \right)},$$ (17)

with ε = 10^−8^. Metrics are computed per session on the test segment and then macro-averaged over 23 sessions.

#### 3.7.3 Aggregation

For MPD-DF main tables, each seed is first averaged over 50 LOSO folds, then mean±std is reported over seeds 2026–2030. For SEED-VIG, sessions are macro-averaged first, then multi-seed mean±std is reported.

### 3.8 Ethics statement

This study is a secondary analysis of two publicly available, fully anonymized datasets (SEED-VIG and MPD-DF); no new human-participant data were collected. Ethical approval for the original data collection was obtained by the respective dataset providers (MPD-DF: Fudan University Ethics Committee, approval No. FE231561). The present secondary analysis did not require additional ethical approval. All participants in the original MPD-DF study provided written informed consent (including data-sharing terms). SEED-VIG is distributed under a public license agreement for academic research use.

## 4 Results

### 4.1 Preliminary analysis of the main feature setting

The main experiments use ROI×α/β (*K* = 8) as input compression. This choice is driven by residual-field and region–band sensitivity observations on MPD-DF relative to an early personal spectral reference, not a claim of unique optimality. Topography and sensitivity analyses below are MPD-DF-only (SEED-VIG is continuous regression without comparable discrete early-state contrasts); cross-corpus usability of the shared ROI×α/β compression is supported by main results in section “4.2 Main results.”

Using the mean spectrum over the opening *L* = 60*s* as reference, we examine scalp maps of relative residuals *d* = *F*_*t*_−μ[μ as in Equation 3. Maps use channel×band residuals directly –the same “subtract early personal spectral reference” setting as the ROI×band-pooled residual in Equation 5, differing only in whether pooling is applied.

Eleven subjects with sufficient mild-fatigue (L1) samples in both an early period (≈0−0.5*h*) and a peak period (≈1.0−1.25*h*) were analyzed. α residuals in early alert and early L1 still form a globally negative field below baseline with highly similar spatial structure (whole-map cosine ≈0.96; difference-map RMS ≈0.053). By peak L1, parieto-occipital residuals reverse from negative to positive; cosine similarity of the α spatial field versus early L1 drops to ≈0.05, with difference energy dominated by α and accompanying β elevation (Figure 3). Thus: early alert and mild fatigue are nearly isomorphic in residual fields; separable fatigue patterns emerge only later.

**FIGURE 3:**
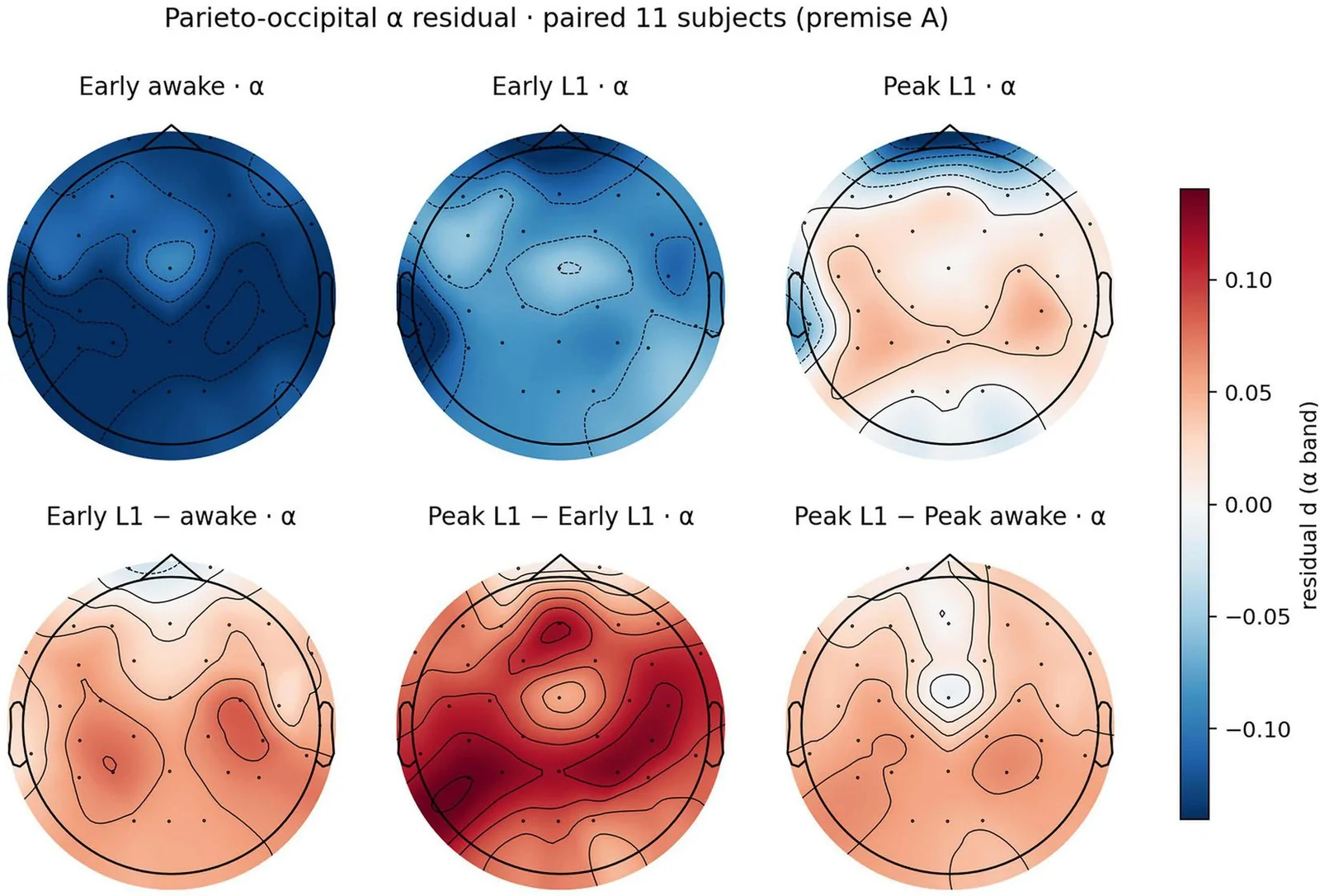
α residual topographies relative to the early personal spectral reference (paired *n* = 11). Top: early alert, early L1, and peak L1; bottom: corresponding difference maps. Color scale is residual *d*; red/blue indicate above/below the early baseline.

This observation indicates that the short opening window is unsuitable as a decision period and is better used to estimate the early personal spectral reference μ. Supervision and readout are therefore placed on subsequent residual trails for *t*≥*L*, with final decisions driven by slow-drift deviations relative to μ.

Projecting residual hotspots onto a 10–20 montage, salient channels concentrate in posterior–lateral occipital, parietal, and temporal regions and align with α (and locally β) (Figure 4). Aggregating channels by anatomical ROI while retaining α/β yields compact tokens while preserving space–band structure (main *K* = 8).

**FIGURE 4:**
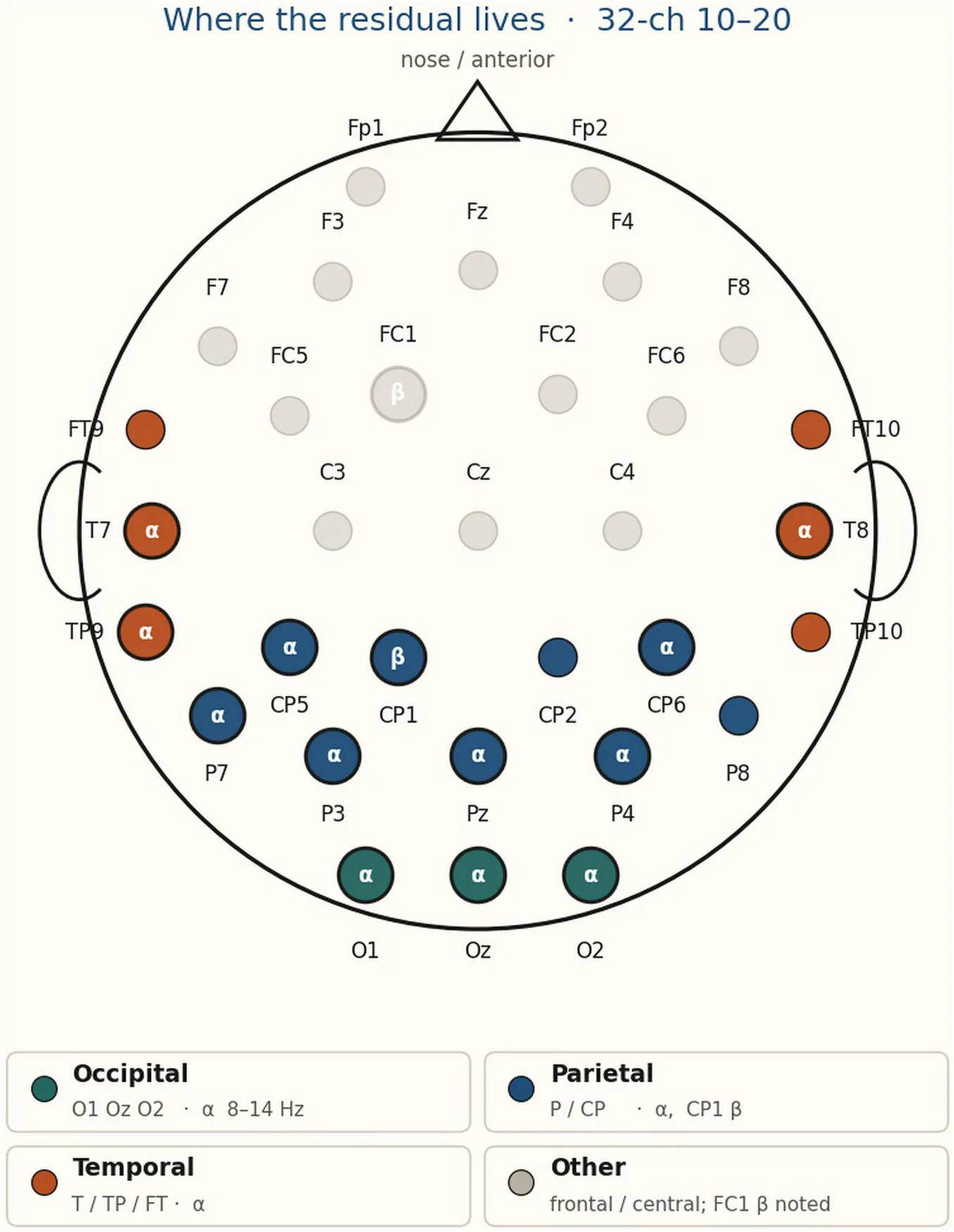
Residual hotspots and ROI placement (32 channels, 10–20). Occipital / parietal / temporal α (and local β) are more prominent.

On a larger sample (effective *n* = 47), taking each individual’s most sensitive region by ∑|Δ*logBP*|, occipital sites are relatively common (≈36%) but not majority; frontal and parietal still account for ≈26% and 21% (Figure 5). There is no single region×band fingerprint covering most people; we therefore treat ROI×α/β pooling as an engineering dimensionality-reduction prior shared across subjects –not a claim of physiological optimality or explicit cross-subject alignment. Hard-coding this prior to *K* = 8 controls input dimension and parameter count under a single causal block (*n*_*b*_ = 1); feeding full channel×band would raise capacity and overfit more easily under LOSO. Learnable attention for adaptive region selection is left to future work. This analysis motivates using the opening window solely to estimate μ; the following sections test whether the design yields task-level gains under standard classification/regression metrics.

**FIGURE 5:**
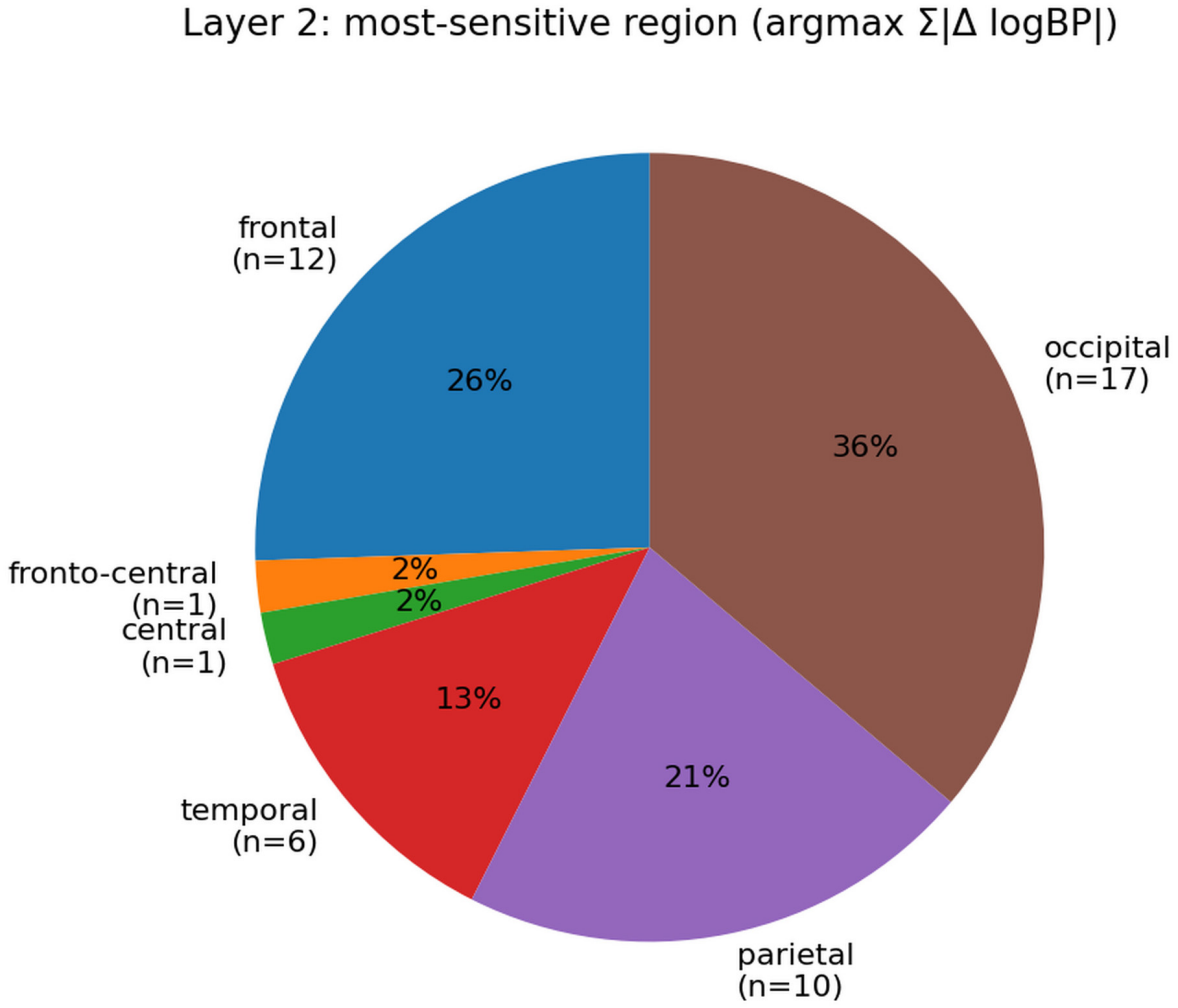
Distribution of individually most sensitive regions (*argmax*∑|Δ*logBP*|, effective *n* = 47).

### 4.2 Main results

#### 4.2.1 Performance comparison and analysis

Tables 3, 4 summarize matched-protocol performance on MPD-DF classification and SEED-VIG regression.

**TABLE 3:** MPD-DF all-50 LOSO (*t*≥*L*; primary metric Macro-F1).

Method	Macro-F1	Acc	Precision	Recall
SVM-RBF	0.565 ± 0.010	0.726 ± 0.011	0.639 ± 0.010	0.645 ± 0.004
MLP	0.574 ± 0.014	0.719 ± 0.012	0.669 ± 0.013	0.655 ± 0.006
ESTCNN	0.550 ± 0.021	0.690 ± 0.018	0.632 ± 0.012	0.629 ± 0.008
VIGNet	0.568 ± 0.028	0.718 ± 0.028	0.659 ± 0.023	0.639 ± 0.020
DGCNN	0.566 ± 0.024	0.717 ± 0.025	0.671 ± 0.017	0.653 ± 0.020
EEGNet	0.587 ± 0.012	0.733 ± 0.014	0.668 ± 0.014	0.658 ± 0.015
DBN	0.574 ± 0.012	0.749 ± 0.009	0.680 ± 0.009	0.637 ± 0.006
GCBNet	0.572 ± 0.013	0.725 ± 0.015	0.672 ± 0.020	0.649 ± 0.012
CDCN	0.514 ± 0.019	0.683 ± 0.023	0.642 ± 0.018	0.603 ± 0.011
EEG-Deformer	0.577 ± 0.012	0.734 ± 0.017	0.671 ± 0.022	0.656 ± 0.012
TSception	0.573 ± 0.004	0.732 ± 0.006	0.677 ± 0.014	0.639 ± 0.007
LDGCN	0.581 ± 0.019	0.736 ± 0.019	0.659 ± 0.017	0.654 ± 0.015
CORAL	0.569 ± 0.027	0.713 ± 0.022	0.659 ± 0.025	0.641 ± 0.027
DANN	0.559 ± 0.011	0.720 ± 0.018	0.655 ± 0.019	0.629 ± 0.010
MSMDA	0.597 ± 0.011	0.786 ± 0.006	0.695 ± 0.017	0.648 ± 0.016
**Ours**	**0.655 ± 0.005**	**0.807 ± 0.004**	**0.713 ± 0.006**	**0.699 ± 0.003**

Best in bold, second best underlined. External baselines are matched-protocol absolute-spectrum frame decoders (mean ± std over seeds 2026–2030; not matched capacity). Matched long-sequence encoding without early −μ (w/o Early Baseline) is reported in section “4.3 Ablation studies,” not here.

**TABLE 4:** SEED-VIG 5Bands / 2 Hz comparison (RMSE↓ / MAPE(%)↓; mean ± std over seeds 2026–2030).

Method	5Bands RMSE	5Bands MAPE	2 Hz RMSE	2 Hz MAPE
SVR	0.261 ± 0.003	86.7 ± 2.7	0.226 ± 0.005	82.3 ± 2.6
ESTCNN	0.267 ± 0.014	78.9 ± 3.1	0.315 ± 0.022	97.0 ± 9.3
VIGNet	0.225 ± 0.007	74.2 ± 3.2	0.239 ± 0.006	76.6 ± 1.3
DGCNN	0.258 ± 0.010	78.1 ± 2.4	0.240 ± 0.007	76.3 ± 2.3
EEGNet	0.250 ± 0.010	74.4 ± 3.1	0.263 ± 0.012	81.4 ± 4.5
DBN	0.260 ± 0.012	83.2 ± 6.1	0.243 ± 0.007	79.6 ± 3.9
GCBNet	0.241 ± 0.002	72.5 ± 4.0	0.233 ± 0.010	68.1 ± 4.9
EEG-Deformer	0.250 ± 0.014	79.3 ± 11.0	0.244 ± 0.012	78.9 ± 3.9
TSception	0.284 ± 0.015	89.1 ± 5.7	0.261 ± 0.020	82.7 ± 5.3
LDGCN	0.245 ± 0.012	75.6 ± 3.8	0.251 ± 0.011	78.6 ± 6.9
CDCN	0.240 ± 0.006	78.9 ± 3.0	0.251 ± 0.010	81.6 ± 6.0
CORAL	0.257 ± 0.014	75.7 ± 6.7	0.269 ± 0.019	77.5 ± 5.6
DANN	0.255 ± 0.020	81.6 ± 4.4	0.264 ± 0.013	78.1 ± 5.9
MSMDA	0.273 ± 0.017	82.2 ± 10.9	0.278 ± 0.006	86.7 ± 3.5
Ours	**0.209 ± 0.005**	**55.8 ± 1.4**	**0.200 ± 0.003**	**53.8 ± 1.7**

Best in bold, second best underlined. External baselines are matched-protocol absolute-spectrum frame decoders (not matched capacity: current frame *F*_*t*_ only, no long trail). Matched long-sequence encoding without early −μ (w/o Early Baseline) is reported in section “4.3 Ablation studies,” not here.

MPD-DF (Table 3). Under 50-subject LOSO, the proposed method at seed 2026 achieves Macro-F1/Acc/Precision/Recall of 0.660/0.809/0.716/0.703; five-seed (2026–2030) means are 0.655 ± 0.005/0.807 ± 0.004/0.713 ± 0.006/0.699 ± 0.003. The method outperforms all absolute-spectrum controls in the table (best EEGNet 0.587) and three representative domain-alignment/transfer baselines (MSMDA 0.597, CORAL 0.569, DANN 0.559), indicating that residual trails with state-*e* readout enable cross-subject classification without absolute-spectrum scoring and without domain alignment.

SEED-VIG (Table 4). Under the same LOSO protocol as the baselines and without multi-source transfer (matched protocol, not matched capacity; baselines read the current frame only), with a shared backbone and MLP regression head: 5Bands RMSE/MAPE 0.209 ± 0.005/55.8 ± 1.4; 2 Hz 0.200 ± 0.003/53.8 ± 1.7 (seeds 2026–2030). Both RMSE values beat absolute-spectrum controls in the table: runner-up VIGNet 0.225 ± 0.007 on 5Bands and runner-up SVR 0.226 ± 0.005 on 2 Hz. MAPE is also lowest under both settings (5Bands 55.8 vs runner-up GCBNet 72.5; 2 Hz 53.8 vs runner-up GCBNet 68.1). Overall, early-frozen early personal spectral reference μ plus causal long-sequence residual encoding (readout from *e*) outperforms matched absolute-spectrum baselines and representative domain-alignment/transfer methods on both discrete classification and continuous regression –sharing one backbone and readout interface, with only linear/MLP heads swapped by task. Figure 6 visualizes primary-metric gaps from Tables 3, 4. A matched-backbone control that keeps the same long-sequence encoder but removes early −μ is reported as w/o Early Baseline in section “4.3 Ablation studies” and shows large degradations on both datasets.

**FIGURE 6:**
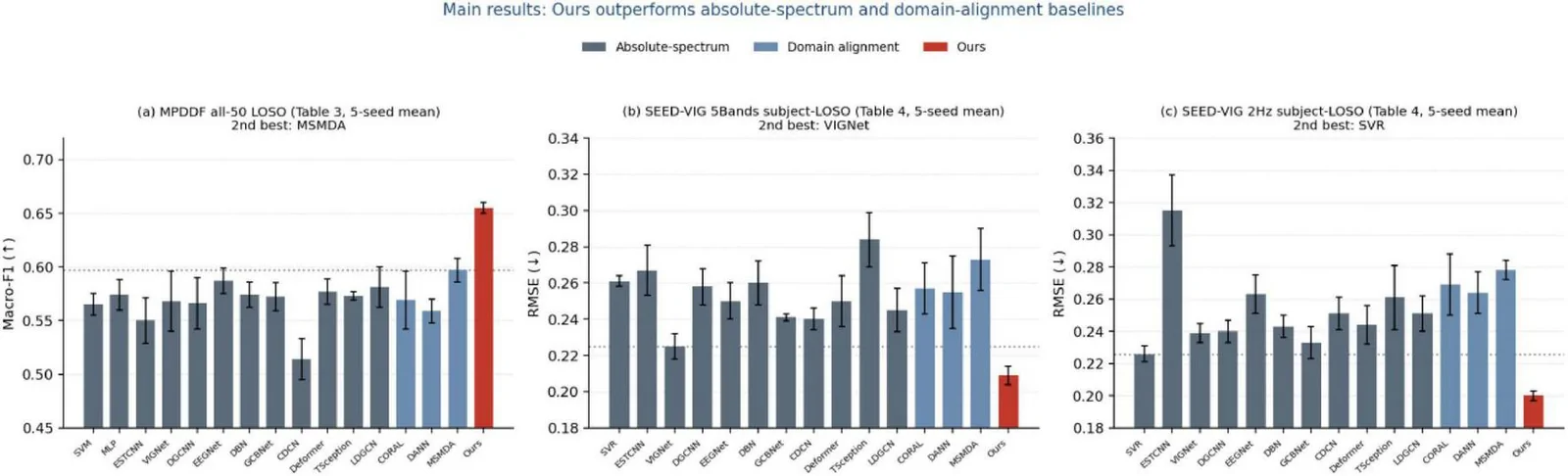
Bar comparison of main results. **(a)** MPD-DF Macro-F1 (Table 3, five-seed mean ± std); **(b)** SEED-VIG 5Bands RMSE; **(c)** SEED-VIG 2 Hz RMSE (Table 4, five-seed mean ± std). Red bars: proposed method; gray dashed lines: runner-up baselines (named in subplot titles).

#### 4.2.2 MPD-DF main comparison table

Table 3 summarizes the MPD-DF comparison under the all-50 LOSO protocol (seeds 2026–2030). Our method achieves Macro-F1 0.655, outperforming the best absolute-spectrum baseline (EEGNet, 0.587) and the best domain-alignment baseline (MSMDA, 0.597). Matched long-sequence encoding without early μ (w/o Early Baseline) is reported in Section 4.3, not here.

#### 4.2.3 SEED-VIG main comparison (5Bands/2 Hz)

Table 4 summarizes the SEED-VIG 5Bands/2 Hz comparison under the subject-LOSO protocol (seeds 2026–2030). Our method achieves RMSE 0.209/0.200, outperforming all matched-protocol baselines.

### 4.3 Ablation studies

Taking the full method as reference, we remove or replace one component at a time to examine: (1) early subtraction of μ; (2) the full long-sequence residual encoding block of section “3.3 Causal long-sequence residual encoding;” (3) readout from updated state *e* versus instantaneous residual *d*_*now*_; (4) SEED regression-head capacity (Linear vs MLP128; see section “3.4 Readout Head).” (5) sensitivity to opening freeze length *L* [section “4.3.5 Freeze-Length *L* Sensitivity (MPD-DF)];” (6) sensitivity to causal encoding window *T* [section “4.3.6 Causal Encoding Window *T* Sensitivity (MPD-DF)].” Table 5 summarizes the first three structural ablations (on/off only); head-capacity controls appear later in this section, using the same metric columns as the SEED ablation table.

**TABLE 5:** Ablation configurations (relative to the full method).

Variant	Early −μ	Long-seq. residual enc.	Readout	Description
w/o Early Baseline	×	✓	updated *e*	No −μ; encode absolute spectral trails
w/o Trail Encoding	✓	×	updated *e*	Encoding off; still read initialized *e*
w/o State Readout	✓	✓	*d*_*now*_	Encoding may run; readout uses *d*_*now*_ only
**Ours (Full)**	✓	✓	**updated** *e*	**Encoding on; readout from updated** *e*

“Long-sequence residual encoding” denotes the section “3.3 Causal long-sequence residual encoding” block (TokenMix, TimeMix, Causal Persist, and state update). w/o State Readout differs from Full only in the readout signal; *d*_*now*_ is the instantaneous residual *d* of (5), fixed before encoding and independent of encoding outputs. w/o Trail Encoding differs from Full in whether residual accumulation occurs inside the window: the former turns encoding off and reads initialized *e* without within-window accumulation; the latter accumulates inside the window and then reads updated *e*. Neither inherits state across supervised times –both re-initialize *e* at each supervised time. Regression-head capacity (MLP128→Linear on SEED) does not change this table’s three switches and is reported separately later in this section. Bold text highlights our proposed method (Ours).

#### 4.3.1 Impact of early frozen reference

Removing early −μ sharply degrades both ends: MPD-DF Macro-F1/Acc fall from 0.660/0.809 to 0.590/0.760; SEED-VIG 5Bands/2 Hz RMSE/MAPE rise from 0.209/55.8 and 0.200/53.8 to 0.255/69.0 and 0.245/68.3 (Tables 6, 7). Absolute spectral patterns alone cannot reliably separate “who” from “whether fatigued;” an early frozen window is needed to estimate the early personal spectral reference. Collapse is of similar magnitude under both feature settings, so the benefit of −μ is not tied to a particular band partition. Note that “near-isomorphism of early two states” in section “4.1 Preliminary analysis of the main feature setting” holds only for MPD-DF discrete labels; SEED-VIG continuous PERCLOS regression has no comparable discrete early contrast, and its opening window cannot *a priori* be treated as a pure alertness anchor. Yet −μ remains effective on SEED-VIG even when opening PERCLOS is nonzero, further supporting that the core role of −μ is removing individual offset, not depending on an “opening must be alert” premise.

**TABLE 6:** MPD-DF ablations (Macro-F1 / Acc / Precision / Recall; seeds 2026–2030, 50 folds).

Variant	Macro-F1	Acc	Precision	Recall
w/o Early baseline	0.583 ± 0.008	0.767 ± 0.011	0.679 ± 0.017	0.648 ± 0.005
w/o Trail encoding†	0.625 ± 0.003	0.799 ± 0.002	0.750 ± 0.009	0.652 ± 0.003
w/o State readout	0.615 ± 0.008	0.794 ± 0.004	0.729 ± 0.017	0.649 ± 0.008
**Ours (Full)**	**0.655 ± 0.005**	**0.807 ± 0.004**	**0.713 ± 0.006**	**0.699 ± 0.003**

† Corresponds to Table 5 with encoding off and readout still from *e*. w/o State Readout keeps encoding on but switches readout to *d*_*now*_. MPD-DF ablations are multi-seed (2026–2030) illustrations; interpret relative gaps to Full. SEED reports five-seed mean ± std. At initialization ψ≡0 implies *e*≡*d*, so w/o Trail Encoding and w/o State Readout diverge only after training, depending on whether encoding rewrites the trail. Bold text highlights our proposed method (Ours).

**TABLE 7:** Matched-structure SEED-VIG ablations (RMSE↓ / MAPE(%)↓; seeds 2026–2030).

Variant	5Bands RMSE	5Bands MAPE	2 Hz RMSE	2 Hz MAPE
w/o Early Baseline	0.255 ± 0.005	69.0 ± 1.9	0.245 ± 0.002	68.3 ± 2.2
w/o Trail Encoding	0.218 ± 0.005	59.8 ± 1.6	0.212 ± 0.004	59.5 ± 1.7
w/o State Readout	0.220 ± 0.005	59.8 ± 2.3	0.208 ± 0.004	57.6 ± 2.9
**Ours (Full)**	**0.209 ± 0.005**	**55.8 ± 1.4**	**0.200 ± 0.003**	**53.8 ± 1.7**

Bold text highlights our proposed method (Ours).

#### 4.3.2 Long-sequence residual encoding

Disabling the full section “3.3 Causal long-sequence residual encoding” long-sequence residual encoding block degrades both classification and regression by similar relative amounts. On MPD-DF, Macro-F1 falls from 0.660 to 0.626 (about −5.2% relative); under subject-LOSO on SEED-VIG, 5Bands RMSE rises from 0.209 to 0.218 and 2 Hz from 0.200 to 0.212 (about +4.3%/+6.0% relative; Tables 6, 7). Sequence accumulation therefore benefits both tasks with comparable magnitude, without a clear task asymmetry. This direction is consistent with section “4.3.3 Readout: **u**pdated state *e* vs instantaneous residual *d*_*now*_": early −μ is indispensable on both tasks, and sequence accumulation contributes on both classification and regression.

#### 4.3.3 Readout: updated state *e* vs instantaneous residual *d*_*now*_

w/o State Readout switches readout to *d*_*now*_ (encoding may still run, but updated *e* is unused). Here *d*_*now*_ is exactly *d* in (5): the pre-encoding pooled residual of the current frame *F*_*t*_ (trail end) relative to μ, not the last frame of *X* after TokenMix/TimeMix. *e* Relative to Full: MPD-DF Macro-F1/Acc drop from 0.660/0.809 to 0.599/0.788 (about −9.2% relative); SEED-VIG 5Bands/2 Hz RMSE are 0.220/0.208 versus Full 0.209/0.200 (about +5.3%/+4.0% relative). Proportionally, classification degrades more than regression, indicating stronger dependence of classification on updated *e*, whereas instantaneous residuals still yield a modest gain on regression with a smaller gap. The distinction from w/o Trail Encoding is: the latter turns off the entire encoding block and reads initialized *e* [started from instantaneous residual *d* in Equation 5]; the former still runs encoding and updates *e* but reads *d*_*now*_. Combined with section “3.6 Experimental setup:” SEED-VIG regresses PERCLOS every second for *t*≥*L*, whereas MPD-DF samples discrete labels every 10 *s* –differences in supervision density and decision granularity help explain this readout sensitivity.

#### 4.3.4 Regression-head capacity (Linear vs MLP)

Tables 5–7 fix the SEED regression head as MLP128 (section “3.4 Readout head).” To test necessity of this capacity, under the same backbone, same readout *e*, and same evaluation protocol, we replace only the head with *Linear*(*K*→1) (Linear Head), compared with same-seed Ours (Full). Table 8 uses the same columns as Table 7 (RMSE↓/MAPE(%)↓).

**TABLE 8:** SEED-VIG head capacity (seeds 2026–2030; Full backbone).

Variant	5Bands RMSE	5Bands MAPE	2 Hz RMSE	2 Hz MAPE
Linear head	0.229 ± 0.005	66.1 ± 3.7	0.220 ± 0.005	65.5 ± 1.9
**Ours (Full, MLP128)**	**0.209 ± 0.005**	**55.8 ± 1.4**	**0.200 ± 0.003**	**53.8 ± 1.7**

Only the readout head is replaced; the three switches in Table 5 remain identical to Full. Results are five-seed (2026–2030) mean ± std, matching Table 7. Relative to Full, Linear Head increases 5Bands / 2 Hz RMSE by about 0.020 / 0.020 and MAPE by about 10.3 / 11.7 percentage points. Bold text highlights our proposed method (Ours).

Continuous PERCLOS estimation thus requires nonlinear readout capacity; MPD-DF classification already uses a linear head and needs no parallel control. We therefore retain task-asymmetric heads rather than forcing Linear on both ends.

#### 4.3.5 Freeze-length *L* sensitivity (MPD-DF)

The main setting uses *L* = 60. To test whether “longer is better,’ ’ we scan *L* ∈ {1,5,10,20,30,60,90,120}*s* under the same structure with five seeds (2026–2030); supervision and evaluation are unified to start at *t*≥120*s* so that eligible time sets are comparable across freeze lengths (other hyperparameters match the main method). Table 9 examines the opening freeze length *L* used to estimate the early personal spectral reference μ, not the causal encoding window *T*; *L* does not control how much history the encoder sees. Table 9 therefore reports absolute Macro-F1 under this unified *t*≥120*s* protocol and must not be compared with Table 3 (where supervision starts at *t*≥*L* = 60*s*). Table 9 is only for ranking *L* within the same scan.

**TABLE 9:** Freeze-length sensitivity on MPD-DF (5 seeds 2026–2030, 50 folds; unified *t*≥120*s* –not comparable to Table 3).

L (s)	Macro-F1	Acc	Precision	Recall
1	0.656 ± 0.006	0.811 ± 0.004	0.713 ± 0.005	0.693 ± 0.006
5	0.657 ± 0.006	0.812 ± 0.004	0.712 ± 0.005	0.694 ± 0.006
10	0.654 ± 0.006	0.810 ± 0.004	0.710 ± 0.005	0.694 ± 0.006
20	0.652 ± 0.006	0.808 ± 0.004	0.708 ± 0.005	0.694 ± 0.006
30	0.650 ± 0.004	0.807 ± 0.003	0.706 ± 0.002	0.693 ± 0.004
60	0.655 ± 0.007	0.805 ± 0.003	0.714 ± 0.005	0.698 ± 0.007
90	0.654 ± 0.010	0.804 ± 0.004	0.718 ± 0.005	0.697 ± 0.010
120	0.654 ± 0.010	0.806 ± 0.005	0.721 ± 0.004	0.694 ± 0.007

This table examines opening freeze length *L* used to estimate μ, not causal encoding window *T*; *L* does not control encoder-visible history.

Gaps among *L* settings within 1–120 s are only about 0.007; performance is insensitive, and extending to 90/120 yields no stable gain. This supports a short opening freeze rather than “as long an opening history as possible.’ ’ Performance at *L* = 1*s* already matches longer windows, indicating that the personal spectral reference can be estimated from a very short opening snippet.

#### 4.3.6 Causal encoding window *T* sensitivity (MPD-DF)

The main setting uses *T* = 600*s*. To test whether “longer is better,’ ’ we scan *T* ∈ {10,20,30,50,60,300,600}*s* under the same structure with five seeds (2026–2030); supervision and evaluation follow the main protocol (supervision from *t*≥*L* = 60*s*, every 10 s on MPD-DF), with other hyperparameters matching the main method. *T* is the causal encoding-window length and controls encoder-visible history; it is distinct from the opening freeze length *L* scanned in Table 9 (used to estimate μ): *L* reflects stability of the individual spectral intercept, whereas *T* reflects how much history the encoder sees. Table 10 reports absolute Macro-F1 under this protocol, with the same supervision start as Table 3.

**TABLE 10:** Causal encoding-window *T* sensitivity on MPD-DF (5 seeds 2026–2030, 50 folds).

T (s)	Macro-F1	Acc	Precision	Recall
10	0.628 ± 0.005	0.799 ± 0.003	0.742 ± 0.010	0.654 ± 0.003
20	0.642 ± 0.004	0.804 ± 0.003	0.714 ± 0.007	0.684 ± 0.004
30	0.656 ± 0.004	0.811 ± 0.003	0.730 ± 0.007	0.692 ± 0.003
50	0.654 ± 0.005	0.809 ± 0.002	0.726 ± 0.006	0.688 ± 0.006
60	0.656 ± 0.004	0.811 ± 0.002	0.725 ± 0.006	0.689 ± 0.006
300	0.655 ± 0.003	0.809 ± 0.002	0.725 ± 0.003	0.690 ± 0.004
600	0.657 ± 0.004	0.810 ± 0.003	0.728 ± 0.007	0.690 ± 0.004

Macro-F1 rises clearly as *T* grows from 10 s to 30 s (0.628→0.656), then plateaus for *T* = 30−600 (0.654–0.657). Causal accumulation therefore needs a lower bound on window length (about 30 s), but not unbounded length; gains come from the causal accumulation mechanism on residuals rather than from the physical length of the window alone.

## 5 Discussion

Cross-subject failure in driving-fatigue EEG is often attributed to “insufficient backbone capacity” or “missing domain alignment.” Our results favor another mechanistic chain: first estimate an early personal spectral reference μ, then decide in residual space. Dual-dataset ablations show large degradation without early –μ –even when SEED-VIG openings are not purely alert. A more general reading of early subtraction is therefore: remove individual spectral offset that is relatively stable across time (analogous to removing a DC component/high-passing the individual intercept), rather than assuming that “what is subtracted must be an alert physiological state.” On MPD-DF, early alert and L1 residuals are nearly isomorphic (section “4.1 Preliminary analysis of the main feature setting”), so that intercept is often near opening alert level and thus resembles an “alertness anchor”; on SEED, even with opening vigilance fluctuations, removing the intercept still improves cross-subject usability. The two views are not contradictory. Regarding spectral evolution, early L1 and alertness on MPD-DF are nearly isomorphic in residual fields, with separable patterns emerging late; SEED-VIG lacks an isomorphic discrete early contrast, and drowsiness-related deviations can be more salient from the opening. Topographic motivation in section “4.1 Preliminary analysis of the main feature setting” is therefore MPD-DF-based and does not imply a shared “early two-state inseparability” narrative on both ends.

Sequence encoding in residual space is the core of the second component. Its necessity has two layers. First, encoding must operate on residuals rather than absolute spectra: absolute spectra mix a temporally stable individual intercept with fatigue-related deviations, and the former often dominates the latter; encoding absolute spectra forces the encoder to handle both, so the individual intercept dominates the learning signal (Table 6, w/o Early Baseline). Subtracting an early-frozen personal spectral reference first leaves the encoder to handle fatigue-related deviations in the residual. Second, residual evidence needs sequence accumulation: single-frame residuals are noisy; causal convolution and weighted pooling inside a causal window aggregate slowly accumulating deviations into a stable representation. Ablations show that turning off the full sequence encoding block lowers MPD-DF Macro-F1 from 0.660 to 0.626 (about −5.2% relative) and raises SEED-VIG 5Bands/2 Hz RMSE from 0.209/0.200 to 0.218/0.212 (about +4.3%/+6.0% relative), with similar degradation on both ends, indicating contribution on both classification and regression.

Sequence encoding contributes similarly to classification and regression, but dependence at readout differs. Replacing readout with instantaneous residual *d*_*now*_ alone lowers MPD-DF Macro-F1 by about 9.2% relative (0.660→0.599), whereas SEED-VIG 5Bands/2 Hz RMSE rise only about 5.3%/4.0% relative (0.209/0.200→0.220/0.208). Classification therefore depends more on updated state *e*, while instantaneous residuals remain relatively adequate on the regression end. This difference aligns with supervision density and decision granularity: SEED-VIG provides continuous PERCLOS every second after *t*≥*L*, so dense supervision makes post-−μ instantaneous residuals sufficient for regression; MPD-DF provides discrete labels every 10 s, so single-frame residuals are unstable and sequence-state readout is more critical. Temporal-scale modeling should therefore match downstream supervision granularity.

To compare directly with existing domain-alignment methods, we include CORAL, DANN, and MSMDA under the same subject-LOSO protocol. On MPD-DF, the best domain-adaptive baseline MSMDA reaches Macro-F1 0.597, still below our 0.655; CORAL and DANN reach 0.569 and 0.559. On SEED-VIG, the three baselines yield RMSE about 0.25–0.28, higher than our 0.209/0.200. Our method uses no target-domain data, matching the domain-generalization setting of CORAL/DANN; MSMDA uses unlabeled target data (a stronger setting) yet still underperforms. Under subject-LOSO, early personal spectral reference with causal sequence residual encoding therefore outperforms these representative baselines without relying on domain alignment. We do not claim domain alignment is ineffective; rather, beyond absolute-spectrum decisions, residual-space decoding from an early-frozen personal reference offers a feasible path that does not depend on domain alignment.

Despite consistent dual-dataset support for the above mechanisms, several expected weaknesses and limitations remain.

First, relative to lightweight single-frame decoders (e.g., EEGNet, DBN), the causal sequence encoder increases parameter count and compute, requiring additional trade-offs for deployment on compute-limited in-vehicle edge devices.

Second, relative to domain-alignment methods such as Euclidean alignment, CORAL, and DANN, we rely on an opening window to estimate the personal spectral reference; artifacts, poor electrode contact, or unstable opening state can bias μ –an inherent vulnerability relative to domain-alignment routes.

Third, relative to multi-source specialized-model ensembling (e.g., ATR-EEG), we use a single shared-parameter model that is structurally simpler but may adapt less well to extreme individual differences than multi-source ensembles.

Fourth, task and label settings are coarse: MPD-DF merges fatigue grades 1–4 into binary labels, losing severity information relative to multiclass or ordinal regression; SEED-VIG uses PERCLOS, an ocular proxy for central fatigue with known limitations.

These limitations may introduce several research biases.

- Dataset bias: MPD-DF and SEED-VIG come from specific labs, devices, and driving scenarios; performance on these corpora does not transfer directly to real in-vehicle, multi-vehicle, multi-center environments.
- Subject and scenario bias: sample sizes are limited (50 subjects on MPD-DF; 23 on SEED-VIG), statistical power is constrained, and driving scenarios are relatively homogeneous.
- Comparison bias: main comparisons are matched-protocol but not matched-capacity –baselines read only the current absolute frame, whereas we use reference μ and a residual trail of length *T* –so contributions of residualization versus sequence encoding are primarily isolated by ablations rather than capacity-matched baseline contrasts.
- Label bias: binary merging and PERCLOS proxies may mask continuous severity changes and bias decision boundaries relative to true fatigue state.

For an overview, Table 11 summarizes main findings, supporting evidence, and hypothesis tests by research question.

**TABLE 11:** Summary of research questions, main findings, and hypothesis tests.

Item	Main finding	Tables	Hypothesis
RQ1: Can early personal spectral reference reduce interference from individual differences?	Early personal spectral reference is a critical premise for cross-subject fatigue decisions; removing it sharply degrades both ends	Tables 6, 7	H1 supported
RQ2: Does sequence accumulation beat instantaneous residuals? What causal-window length is needed?	Sequence encoding contributes to both classification and regression; at readout, classification depends more on the updated state; causal windows plateau after moderate length rather than improving indefinitely	Tables 6, 7, 10	H2 supported
RQ3: Are classification and regression consistent?	Encoding contributions are similar on both ends; at readout, classification is more sensitive to the updated state	Tables 6, 7	H3 partially supported
Baseline comparison	Outperforms matched absolute-spectrum baselines and representative domain-alignment/transfer baselines	Tables 3, 4	–

H3 predicted similar dependence on sequence accumulation for classification and regression. Results: encoding degradations are similar (classification ≈−5.2% relative; regression ≈+4.3%–6.0% relative), supporting H3; at readout, classification is more sensitive to the updated state (≈−9.2% relative vs ≈+4.0%–5.3% on regression), not supporting H3. H3 is therefore partially supported.

## 6 Conclusion

We propose a two-component framework –early personal spectral reference and causal sequence residual encoding –for cross-subject slow-drift fatigue decoding. Main conclusions are as follows.

1. An early personal spectral reference estimated from an early frozen window is a critical premise for cross-subject fatigue decoding; its role should be understood as removing an individual slow spectral intercept, not as binding to “opening must be alert.’ ’
2. At the encoding stage, sequence encoding in residual space contributes to both classification and regression with similar magnitude; both tasks can share one backbone. At the readout stage, both tasks benefit from the updated state relative to the instantaneous residual, with stronger dependence on the classification end. Temporal-scale components can be chosen by task. Sensitivity analysis of causal encoding-window length shows that gains rise then plateau with window length rather than growing indefinitely.
3. Without per-driver models or additional domain alignment, the framework outperforms matched absolute-spectrum baselines and representative domain-alignment/transfer baselines on MPD-DF and SEED-VIG.

Limitations and future work: this work uses binary labels and a fixed ROI×α/β dimensionality-reduction prior; future work may introduce multiclass or ordinal regression, adaptive feature selection, and validation on larger multi-center, multi-scenario data. Overall, shared-parameter personalization need not rely on per-driver models, nor is domain alignment the only path.

## Data Availability

Publicly available datasets were analyzed in this study. This data can be found here: MPD-DF: https://doi.org/10.1038/s41597-026-06634-4 (Li et al., Sci. Data, 2026). SEED-VIG: https://doi.org/10.1088/1741-2552/aa5a98 (Zheng & Lu, J. Neural Eng., 2017); distributed under a public license for academic research use.
